# Improving the immunogenicity of HIV-1 envelope trimer vaccines

**DOI:** 10.1186/1742-4690-8-S2-O39

**Published:** 2011-10-03

**Authors:** Rogier W Sanders

**Affiliations:** 1 Weill Medical College of Cornell University, New York, NY, USA

## 

An HIV-1 vaccine that induces protective antibodies remains elusive because a number of factors limit the quantity and quality of the antibodies raised against the HIV-1 envelope glycoprotein complex (Env). We hypothesized that targeting Env vaccines directly to immune cells would improve Env-specific antibody responses. To this end we explored two approaches. First, we fused trimeric Env gp140 at the C-terminus to proteins that can target and activate B cells: CD40 ligand (CD40L), B-cell Activating Factor (BAFF), and A PRoliferation-lnducing Ligand (APRIL). Trimeric Env fused to APRIL, BAFF or CD40L triggered the secretion of IgM, IgG and IgA from human B cells *in vitro.* In particular Env-APRIL induced higher anti-Env antibody responses in rabbits. Env-APRIL was also more efficient at inducing neutralizing responses against various tier 1 viruses. Second, we embedded immunostimulatory proteins within the Env sequence. We replaced the V1V2 domain of Env with granulocyte-macrophage colony-stimulating factor (GM-CSF). Probing with neutralizing antibodies showed that both the Env and GM-CSF components of the chimeric protein were folded correctly. Furthermore, the embedded GM-CSF domain was functional as a cytokine *in vitro.* Mouse immunization studies demonstrated that chimeric Env-GM-CSF enhanced Env-specific antibody and T cell responses compared to wild-type Env. Collectively, these results show that targeting and activation of immune cells using engineered cytokine domains within the protein can improve the immunogenicity of Env subunit vaccines.

